# Significant ground-level ozone attributed to lightning-induced nitrogen oxides during summertime over the Mountain West States

**DOI:** 10.1038/s41612-020-0108-2

**Published:** 2020-01-30

**Authors:** Daiwen Kang, Rohit Mathur, George A. Pouliot, Robert C. Gilliam, David C. Wong

**Affiliations:** 1 Center for Environmental Measurement and Modeling, U.S. Environmental Protection Agency, Research Triangle Park, NC, USA.

## Abstract

Using lightning flash data from the National Lightning Detection Network with an updated lightning nitrogen oxides (NO_x_) emission estimation algorithm in the Community Multiscale Air Quality (CMAQ) model, we estimate the hourly variations in lightning NO_x_ emissions for the summer of 2011 and simulate its impact on distributions of tropospheric ozone (O_3_) across the continental United States. We find that typical summer-time lightning activity across the U.S. Mountain West States (MWS) injects NO_x_ emissions comparable to those from anthropogenic sources into the troposphere over the region. Comparison of two model simulation cases with and without lightning NO_x_ emissions show that significant amount of ground-level O_3_ in the MWS during the summer can be attributed to the lightning NO_X_ emissions. The simulated surface-level O_3_ from a model configuration incorporating lightning NO_x_ emissions showed better agreement with the observed values than the model configuration without lightning NO_x_ emissions. The time periods of significant reduction in bias in simulated O_3_ between these two cases strongly correlate with the time periods when lightning activity occurred in the region. The inclusion of lightning NO_x_ increased daily maximum 8 h O_3_ by up to 17 ppb and improved model performance relative to measured surface O_3_ mixing ratios in the MWS region. Analysis of model results in conjunction with lidar measurements at Boulder, Colorado during July 2014 corroborated similar impacts of lightning NO_x_ emissions on O_3_ emissions estimated for other summers is comparable to the 2011 air quality. The magnitude of lightning NO_x_ estimates suggesting that summertime surface-level O_3_ levels in the MWS region could be significantly influenced by lightning NO_x_.

## INTRODUCTION

Due to its adverse impact on human health^1^ and ecosystem well being,^2^ tropospheric ozone (O_3_) has been at the center of air quality research during the past decades.^3–5^ Ground-level O_3_ is either produced through complex photochemical reactions involving nitrogen oxides (NO_x_) and volatile organic compounds (VOCs),^6^ with both anthropogenic and natural origins, or transported from other locations.^7^ As air quality regulations are tightened and the contribution of anthropogenic sources to tropospheric O_3_ levels is reduced in US and other economically-developed regions, the natural sources becomes more increasingly important.^8–12^

Lightning generates relatively large but uncertain quantities of NO_x_ and thus strongly impacts the composition of trace gases in the troposphere.^13–15^ Globally, lightning is estimated to generate 2–8 Tg N per year^16^ which is considerably smaller than present-day global contributions from anthropogenic (~20.5 Tg N per year) and biomass burning (~5.5 Tg N per year) sources.^17^ Despite the strong influence on troposphere burdens of O_3_ and OH, impacts of lightning NO_x_ emissions on the surface-level air composition and chemistry have been suggested by past studies to be generally small, and thus except for some applications with global models, regional air quality assessments have historically not included lightning NO_x_ emissions.^16^ Uncertainties in lightning NO_x_ emissions are influenced by limitations in the description of space and time variability in lightning frequency and intensity, NO_x_ production rates from lightning flashes, and the vertical distribution and transport of lightning NO_x_ after its production. Since lightning activity is often associated with deep convection in the atmosphere, the lightning flash rate can be parameterized using convection schemes.^18,19^ However, convection schemes in meteorological models generally show low skill in producing the convective precipitation and the lightning distribution.^20,21^ Though many studies report enhancement in surface-level NO_x_ levels following thunderstorm activity, the impacts on surface-level O_3_ are ambiguous and typically complicated by the local photochemical conditions such as the availability of other precursors and sunlight.^22–25^ Regional modeling calculations have shown up to 10 ppb enhancements in simulated instantaneous O_3_ mixing ratios^8,13,23,25^ due to addition of lightning NO_x_.

Heath et al.^26^ demonstrated that assimilation of high-quality lightning data from the national lightning detection network (NLDN) in the weather research and forecast (WRF) model considerably improved the simulation of summertime convective activity and rainfall over the continental United States. In this study, we build upon this prior work^26,27^ by implementing lightning NO_x_ production in the Community Multiscale Air Quality (CMAQ)^28–31^ model based on the NLDN lightning flash data^32,33^ and perform CMAQ simulations driven by the lightning-assimilated WRF outputs with (hereafter denoted NLDN) and without (denoted Base) the NLDN-lightning NO_x_ production.

## RESULTS

**Fig. 1:**
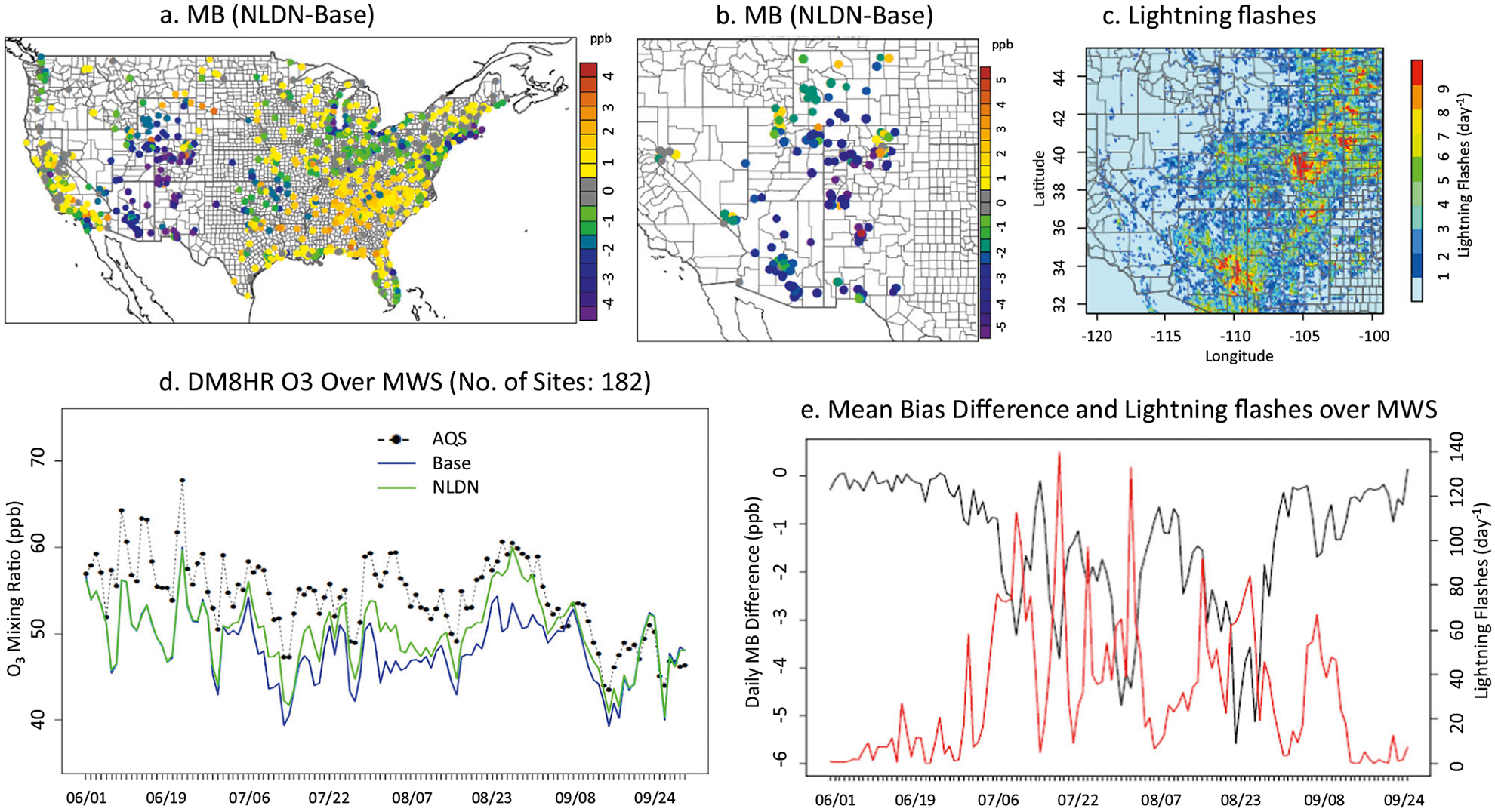
Impact of lightning events on O_3_ production during summer 2011. **a** Mean bias reduction over continental US between simulations with and without lightning NO_x_. **b** Same as **a** but focus on the MWS. **c** The average daily lightning flashes during July 2011. **d** Time series of observed and modeled DM8HR O_3_ mixing ratios. **e** Time series of the mean bias difference between the two simulations and the daily mean lightning flashes.

The model performance for O_3_ predictions, in terms of a particular implementation of lightning NO_x_ production, depends on correct NO_x_ emissions from other sources in the Base model. Uncertainties in specification of precise activity patterns of mobile and industrial sources lead to uncertainties in space and time variability in estimated emissions from these sources that then translate to biases and errors in model predicted O_3_ mixing ratios. The addition of lightning NO_x_ emissions can then result in further deterioration of model performance for predicted O_3_ at many locations such as in the eastern U.S. as illustrated in Fig. 1a, which presents the change in bias in simulated surface daily maximum 8 h (DM8HR) O_3_ between the NLDN and Base simulations. However, significant impact of lightning NO_x_ emissions on improving skill of model predictions of surface O_3_ in the Mountain West States (MWS: Arizona, New Mexico, Nevada, Utah, Colorado, and Wyoming) is illustrated in Fig. 1a, and further emphasized in Fig. 1b. During the summer of 2011 in the MWS region there were over 800 measured DM8HR O_3_ exceedances above 70 ppb as summarized in Table 1. Even though both model simulations with and without lightning NO_x_ underestimated the observed values, the simulation incorporating impacts of lightning NO_x_ reduced the mean bias across the measurement locations (Fig. 1b) significantly during periods with lightning activity (depicted in Fig. 1c). Significant impacts of lightning NO_x_ on day to day variations in domain-mean surface DM8HR O_3_ over the MWS region is also noted in Fig. 1d, which illustrates that lightning NO_x_ emissions increase region-average DM8HR O_3_ by up to 6 ppb and helps reduce the model low-bias. Recent modeling analysis^34^ in the southeast U.S. has speculated that NO_x_ motor vehicle emissions in U.S. National Emission Inventory (NEI) may be overestimated. However, the role of uncertainties associated with other modeled processes as well as the inventory base year used in the analysis, on the magnitude of this overestimation, are not readily apparent (e.g., Simon et al.^10^). The MWS region which is the focus of our analysis is a sparsely populated area and consequently anthropogenic emissions in the region are relatively small. Thus, the relative impacts of uncertainties in anthropogenic NO_x_ on the total NO_x_ burden in the region is also comparatively smaller relative to other regions in the U.S. In recent complementary analyses,^31^ we have comprehensively compared model predicted NO_x_ mixing ratios with observations for different regions, which also indicated that the model has the best performance over MWS for NO_x_ predictions. Thus, the impact of uncertainties in characterizing NO_x_ emissions from other sectors on the inferred impacts of lightning NO_x_ emissions in the MWS region are expected to be negligible.

To further delineate the impact of lightning activity on daily variations in surface O_3_, we examined the correlation between lightning activity and the change in model DM8HR O_3_ bias (Fig. 1e) (see the Supplementary Information (SI) and Supplementary Fig. 5 for detailed calculation of the lightning flashes; here the grid cell increments of ten are used). The time series of DM8HR O_3_ mixing ratios (Fig. 1d) and mean bias difference (Fig. 1e) clearly indicate that the model simulation with lightning NO_x_ (NLDN) performed much better episodically in matching the observed values than the simulation without lightning NO_x_ (Base). Days with highest lightning activity also corresponded to days when the largest improvements in DM8HR O_3_ mean bias were realized through the incorporation of lightning NO_x_ emissions, strongly suggestive of the large role of lightning NO_x_ emissions in modulating surface-level O_3_ mixing ratios in this region. The comparison of the daily average mean bias in DM8HR O_3_ over the AQS sites in the MWS for both the Base and the NLDN cases presented in Supplementary Fig. 3 further illustrates the improvement in model skill in capturing variations in surface DM8HR O_3_ in the region due to inclusion of lightning NO_x_ emissions.

**Fig. 2:**
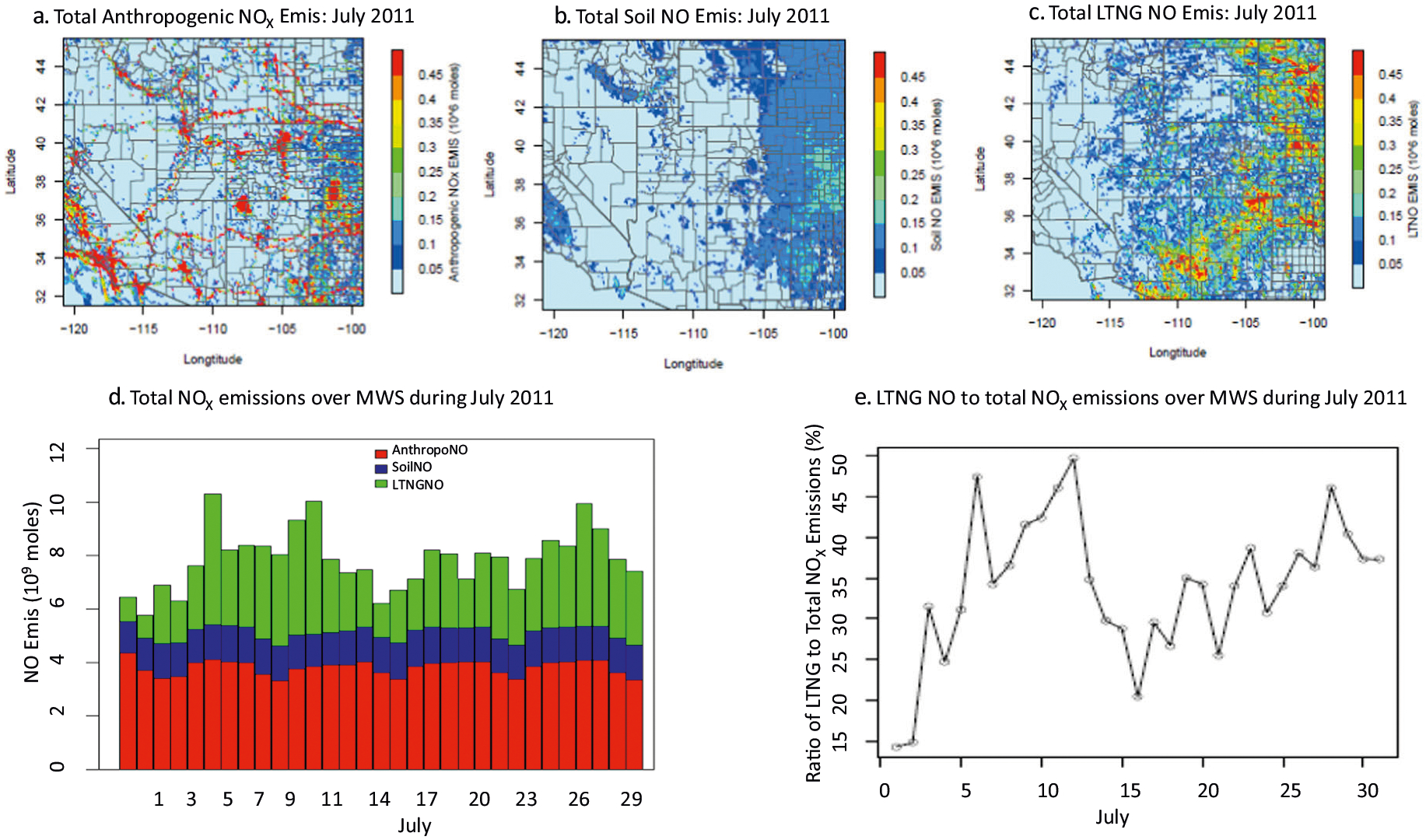
NO_X_ emissions from different sources in time and space during July, 2011. **a** Anthropogenic NO_X_ emissions. **b** Soil NO_X_ emissions. **c** Lightning NO_X_ emissions. **d** Daily total emissions excluding those from the area west of −115° longitude. **e** The daily lightning NO_x_ ratio to the total NO_X_ emissions.

The relative importance of lightning as a source of reactive nitrogen in the troposphere over the MWS region is further highlighted in Fig. 2 which compares the strength of this source with other sources traditionally considered in estimating O_3_ in the region. Figure 2a–c show the spatial distributions of NO_x_ emissions from anthropogenic, soil, and lightning sources over the MWS region for July 2011. Figure 2d presents the relative contributions of these three source categories to daily NO_x_ emissions, while Fig. 2e quantifies the daily fraction of total NO_x_ emissions that can be attributed to lightning. Note, for Fig. 2d, e, we limit the analysis to portions of the MWS region east of the 115°W longitude to minimize the influence of urban centers. As indicated in Fig. 2e, the lightning produced NO_x_ constitutes 15–50% of daily NO_x_ emissions in these remote regions and on average ~30% during July 2011, resulting in the significant impact on surface O_3_ illustrated earlier. The release of lightning NO_x_ in such magnitudes into the atmosphere in this region could also potentially modulate atmospheric nitrogen deposition amounts in the region, and further emphasizes the need for air quality models to include lightning NO_x_ emissions to accurately simulate both atmospheric chemistry and deposition in the MWS region.

**Fig. 3:**
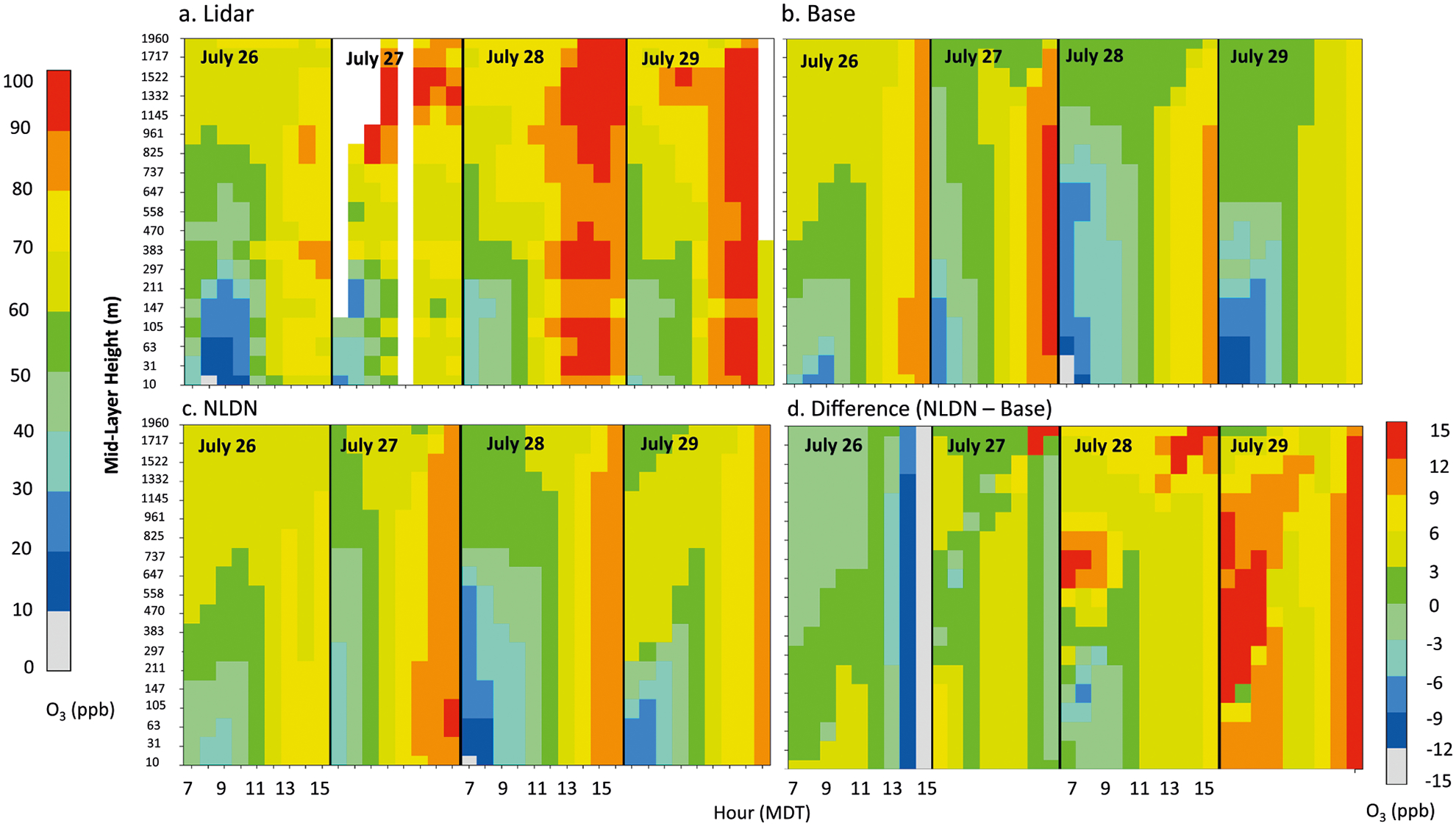
Lidar measured and simulated O_3_ mixing ratios at BAO site. **a** Lidar measurements, **b** base simulations, **c** simulations with NLDN, **d** simulation difference (NLDN – Base). The legend on the left is for **a–c** and the legend at the right bottom is for **d**.

**Fig. 4:**
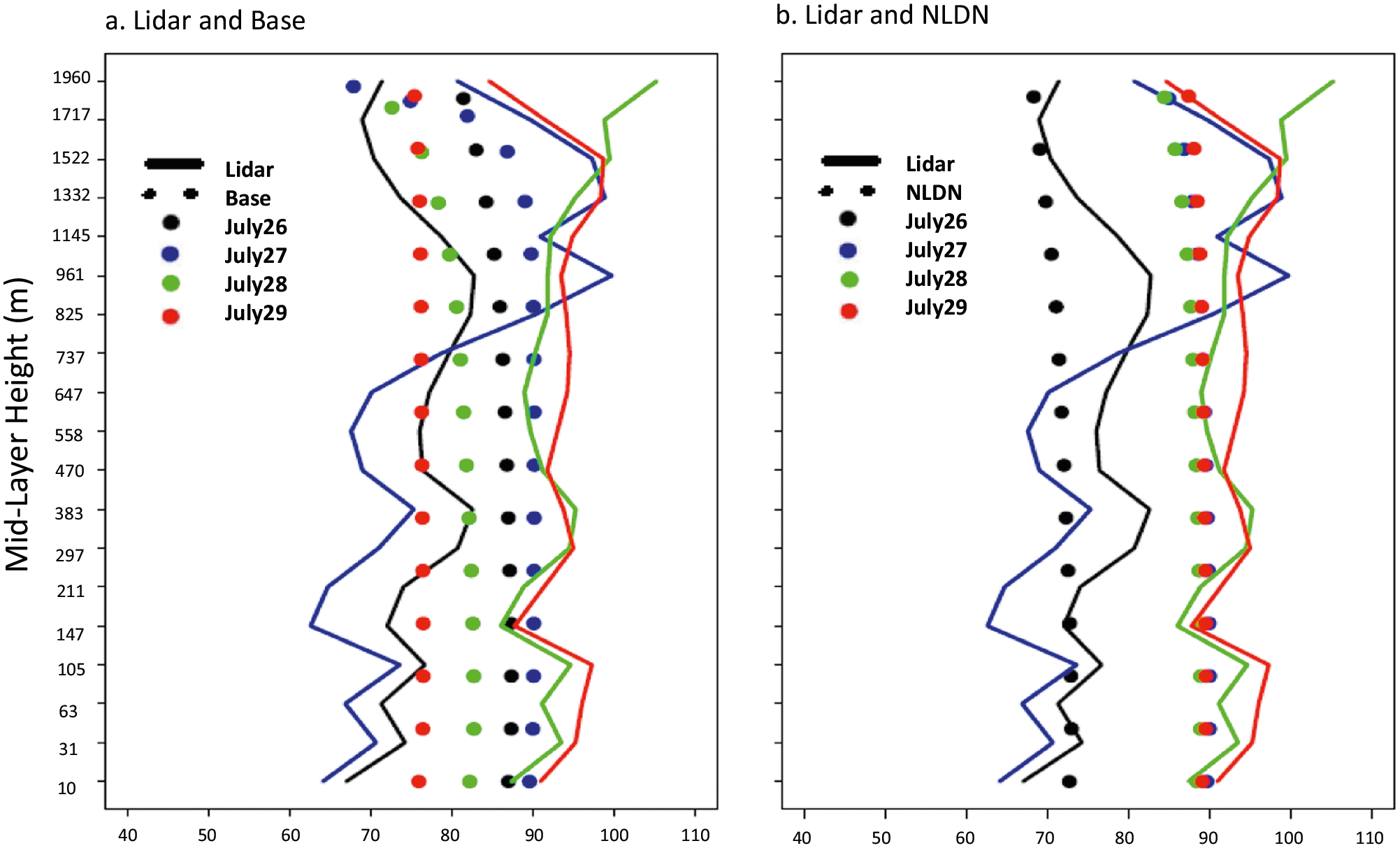
Lidar measured and simulated O_3_ mixing ratios at BAO site. **a** Maximum O_3_ mixing ratios across the layers on each day over the episode (Lidar and Base), and **b** The same as **a**, but for Lidar and NLDN. Note that solid lines represent lidar measurements on different days while the circles are the model values.

The significant impacts of lightning on air quality in the MWS region are however not unique to the summer of 2011. To leverage observations from the 2014 DISCOVER-AQ (Deriving Information on Surface Conditions from COlumn and VERtically Resolved Observations Relevant to Air Quality) field campaign, as a case study, WRF-CMAQ model simulations with (NLDN) and without (Base) lightning NO_x_ emissions were performed for a period encompassing the field study and model results were compared with measurements at the Boulder Atmospheric Observatory (BAO) site. Maximum surface-level O_3_ mixing ratios were observed on July 28th, and the evolution of vertical O_3_ structure inferred from the lidar measurements during 26–29 July 2014 is shown in Fig. 3a. In these illustrations, the observed lidar O_3_ profiles are aggregated to the model vertical grid and temporally averaged to hourly values. Figure 3b shows the corresponding Base model (without lightning NO_x_) simulated values and Fig. 3c shows the corresponding simulated values when lightning NO_x_ is included. Significant discrepancies are noted between the simulated and observed evolution of vertical O_3_ distributions on these days, with the model generally underestimating the development of high O_3_. Also, noted in the observed lidar profiles on most days, is the existence of localized high O_3_ aloft, suggestive of in situ production rather than intrusion of stratospheric air from higher altitudes. Comparison of Fig. 3a, b, c indicates that the simulated evolution of vertical O_3_ distribution and values with lightning NO_x_ (Fig. 3c) matched the observations more closely than the case without lightning emissions. In general, the Base model shows overestimation on July 26th and underestimation on July 28th and 29th, while the simulation with lightning NO_x_ improved the predicted O_3_ values for both these cases, i.e., decreased values on July 26^th^ and increased values on July 28th and 29th. The difference between the two model cases is illustrated in Fig. 3d. The evolution of peak O_3_ over this period is further depicted in Fig. 4a, b that compare model and observed vertical profiles of daily maximum O_3_ mixing ratios for both model cases. From July 26 to 29, the observed peak values increased by over 20 ppb at the surface (from below 70 ppb to above 90 ppb), while the simulated values without lightning NO_x_ decreased by 10 ppb (from 80 to 70 ppb); the simulations changed from more than 10 ppb over-prediction on July 26th to more than 20 ppb under-prediction on July 29th. However, when lightning NO_x_ is included (Fig. 4b), the simulated surface daily maximum O_3_ mixing ratios are much closer to the observed values (compared to the Base case, the simulated O_3_ values decreased from 87 to 72 ppb on July 26th and increased from 76 to 89 ppb on July 29th, respectively). Lightning flash data for this period (see Supplementary Fig. 10) indicate periods of active lightning strikes in the region, especially on July 28th and 29th, further suggesting the contribution of lightning NO_x_ to the observed aloft O_3_ and subsequent contribution to surface enhancements. These changes in model simulated O_3_ mixing ratios during this period in response to the injection of lightning NO_x_ emissions further indicate the non-linear relationship between O_3_ and its precursors. Depending on the location and timing of its injection, lightning NO_x_ emissions can either produce or destroy O_3_. In proximity of the lightning location, O_3_ is chemically consumed due to titration by the freshly emitted NO. In contrast, when NO_x_ from lightning is transported to locations downwind of the lightning flash, in the presence of sunlight it reacts with VOCs in the surrounding air to produce O_3_. Note that the uniform distribution of simulated O_3_ values under about 2 km shown in Fig. 4 and Supplementary Fig. 11 is due to the fact that maximum O_3_ values typically occur during early afternoon hours when the planetary boundary layer (PBL) is well mixed.

**Fig. 5:**
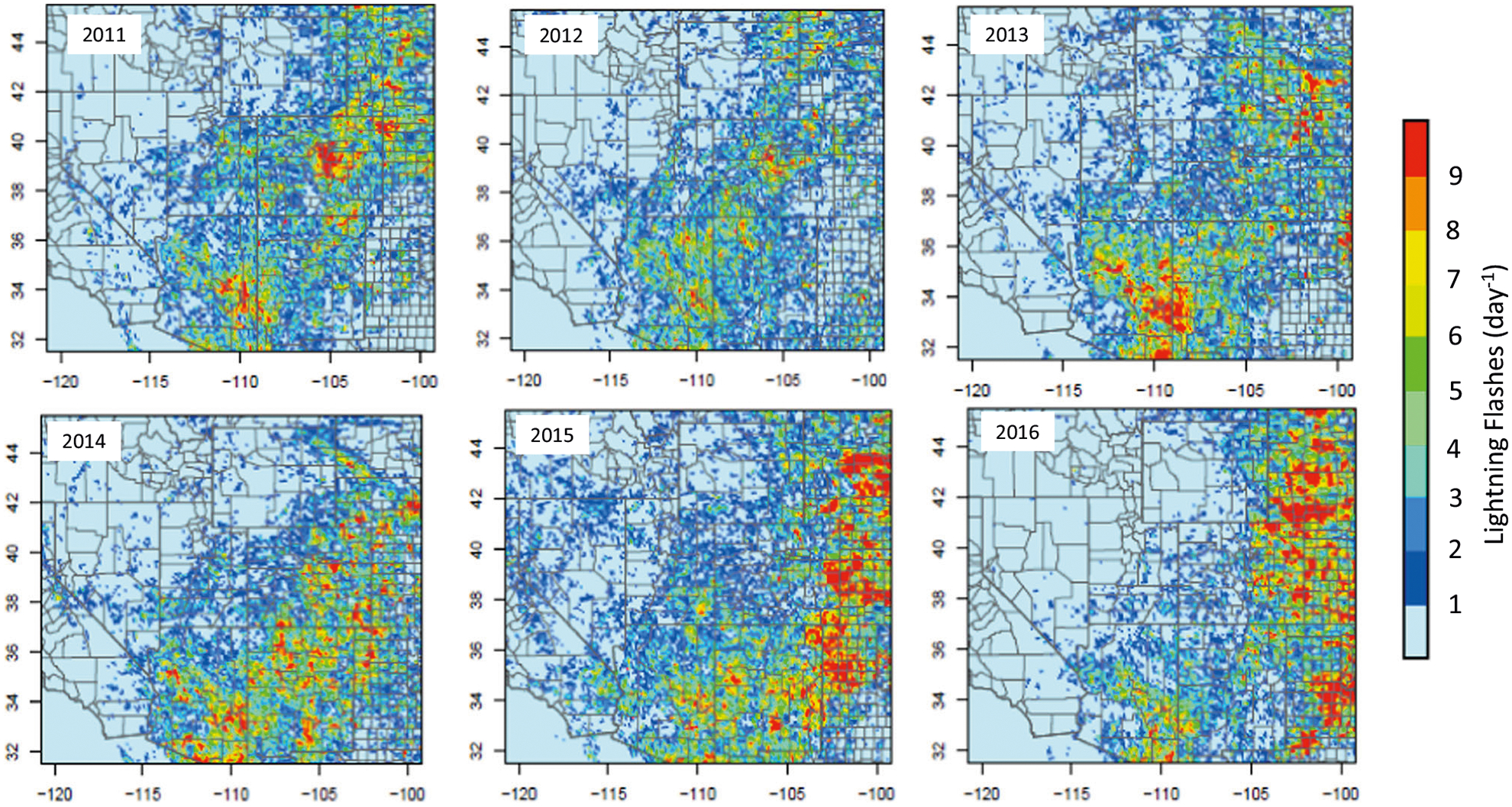
Daily mean lightning flashes over MWS during July from 2011 to 2016.

**Fig. 6:**
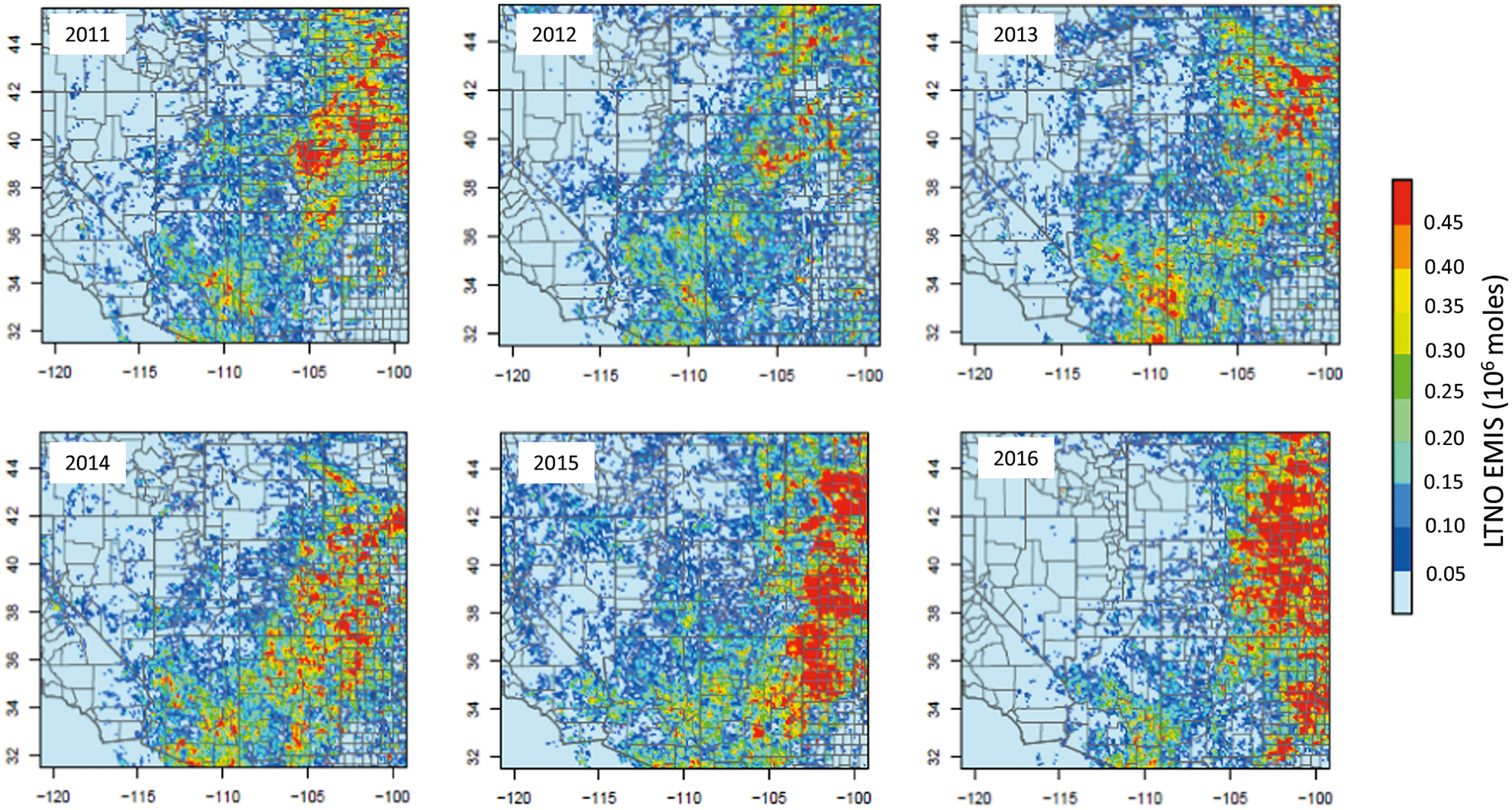
NO_x_ emissions from lightning in July from 2011 to 2016 over MWS.

The importance of lightning NO_x_ as a modulator of tropospheric chemistry in the MWS region is further illustrated in Fig. 5 which presents the observed daily mean lightning flashes across the MWS region for the month of July for the years 2011 through 2016 (similar patterns are also observed for August as illustrated in the SI). Figure 6 shows the estimated NO_x_ emissions released due to these lightning flash rates using the NO_x_ production algorithm implemented in CMAQ.^30,31^ Similar intensity of lightning activity occurs every summer (Fig. 5) resulting in comparable amounts of lightning NO_x_ emissions released into the atmosphere in the MWS region suggesting that summertime O_3_ levels in the region could be significantly modulated by this natural source.

## DISCUSSION

With the most up-to-date lightning NO_x_ production schemes and the vertical distribution algorithm in CMAQ as described and evaluated in recent publications,^30,31^ we examined the importance of lightning NO_x_ as a modulator of tropospheric chemistry in the U.S. Mountain West States. During summer, lightning NO_x_ emissions provide a continuous NO_x_ input to the airshed over the remote MWS region with average monthly contributions to the total NO_x_ budget of 30%. As a result, significant impact on O_3_ air quality is both observed and confirmed through analysis of detailed model simulations with and without lightning NO_x_ emissions and their detailed comparisons with available routine network and specialized field campaign observations of surface and aloft O_3_ in the region.

As revealed by the detailed model calculations for summer 2011, lightning NO_x_ can contribute up to 21 ppb (or 43%) of the hourly values of surface-level O_3_ in the region. Given the comparable level of lightning activity in other years, similar levels of contribution of lightning to surface O_3_ across the region can be expected. More importantly, as technological advances and regulatory measures reduce anthropogenic NO_x_ emissions in the region, the relative contributions of lightning NO_x_ to O_3_ levels in the region may increase. Consequently, accurate quantification of lightning NO_x_ emissions, its contribution to background O_3_ levels, and its role in modulating air quality in the region will become increasingly important.

## METHODS

CMAQ simulations are performed for the April–September 2011 period over a domain encompassing the contiguous United States and discretized with 12 km horizontal grid spacing. The Base model configuration and simulation details are the same as those detailed in Kang et al.^31^ with the CB6 chemical mechanism and emissions input data from the 2011 NEI for 2011 simulations. For simulations during the year 2014, the emissions input data are still based on 2011 NEI with 2014 year-specific emissions for EGU (electric generating units), fire, and biogenic sectors from the three states (Colorado, Utah, and Wyoming) constituting the MWS study region. NO_x_ emissions from electric generating units are based on Continuous Emissions Monitoring and thus are relatively well constrained. The NEI utilizes the MOVES (https://www.epa.gov/moves) modeling system which incorporates detailed information of motor vehicle fleets, miles traveled, fuel information, and the latest emission factors to estimate emissions from this sector. Emissions from wildfires are based on year-specific daily fire-activity from the Hazard Mapping System fire detections and the SMARTFIRE system (http://www.getbluesky.org/smartfire/docs/Raffuse2007.pdf). The lightning-induced NO_x_ is calculated based on the NLDN observed cloud-to-ground (CG) lightning flashes that are gridded to the model grid as hourly total flashes, and intra-cloud (IC) flashes based on the climatological IC-to-CG ratio. The vertical distribution of lightning NO_x_ release follows Kang et al.^30^ The lightning NO_x_ production rate over land-based grid cells is set to 350 moles/stroke (this value is about the middle of the range suggested by recent studies^13^ that have explicitly reported lightning-NO_x_ yields using observational constraints). To evaluate the performance of the two model cases for 2011, we compare simulated hourly and DM8HR O_3_ mixing ratios with corresponding measured values at sites from the air quality system network. To further examine the impact of lightning NO_x_ emissions on atmospheric composition and chemistry we conducted model simulations for the period of the 2014 DISCOVER-AQ field campaign in Colorado and analyzed model time-height O_3_ distributions in conjunction with those inferred from lidar measurements taken by the National Oceanic and Atmospheric Administration’s Earth System Research Laboratory^35,36^ at BAO.

## DATA AVAILABILITY

CMAQ model documentation and released versions of the source code, including all model code used in his study, are available at https://www.epa.gov/cmaq. The data processing and analysis scripts are available upon request. The WRF model is available for download through the WRF website (http://www.wrf-model.org/index.php). All data used to create the tables and illustrations in the manuscript are available at https://zenodo.org/record/3246375. Additional input/output data for CMAQ model utilized for this analysis are available through contact with the corresponding author. The lightning flash observation data in their native form are available for purchase from Vaisala Inc. (https://www.vaisala.com/en/products/systems/lightning-detection).

## Supplementary Material

Supplement1

## Figures and Tables

**Table 1.** Summary of the observed and modeled DM8HR O_3_ mixing ratios in MWS.

Event criteria (ppb)	Number of occurrences (site-day)
MD8HR O_3_ ≥ 70 (observed)	803
Simulated difference (NLDN - Base) ≥ 15	12
Simulated difference ≥ 10	288
Simulated difference ≥ 5	2494
Maximum simulated difference: 17	1
