# Supplementary material for: Significant ground-level ozone attributed to lightning-induced nitrogen oxides during summertime over the Mountain West States
Source: NPJ Clim Atmos Sci. Author manuscript; Available in PMC 2021 Jan 30. (PMC7075249; doi:10.1038/s41612-020-0108-2)
Supplement: Supplement1 [file NIHMS1560789-supplement-Supplement1.pdf]

Supplementary Information for Significant Ground-level Ozone Attributed to Lightning-induced Nitrogen Oxides during Summertime over the Mountain West States

Daiwen Kang, Rohit Mathur, George Pouliot, Robert Gilliam, and David Wong

Center for Environmental Measurement and Modeling, U.S. Environmental Protection Agency, Research Triangle Park, North Carolina, USA

Diurnal profile of hourly O_3 mixing ratios

The hourly mean diurnal O_3 profiles (Supplementary Figure 1) indicate that the impact of lightning NO is not only limited to the daily maximum (or daily maximum 8-hr) O_3 mixing ratios, but rather throughout the day in both July and August suggesting its impact on metrics such as U.S. background O_3 (USBO) that include O_3 formed from natural sources within the U.S.

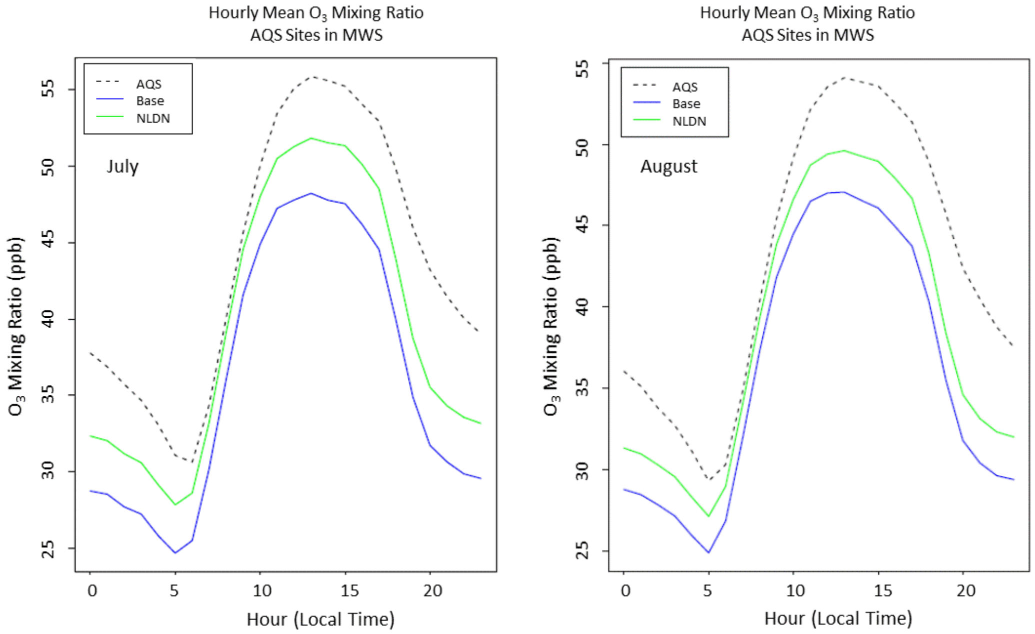

Supplementary Figure 1. Mean hourly diurnal profile of O_3 mixing ratios over the sites in MWS for July and August 2011.

Reduction in simulation errors

The simulation with lighting NO also significantly reduced the errors (Root Mean Square Error, RMSE) as shown in Supplementary Figure 2 over the sites in MWS with more than 90% of the sites in MWS having reduced errors up to 5 ppb (Supplementary Figure 2b).

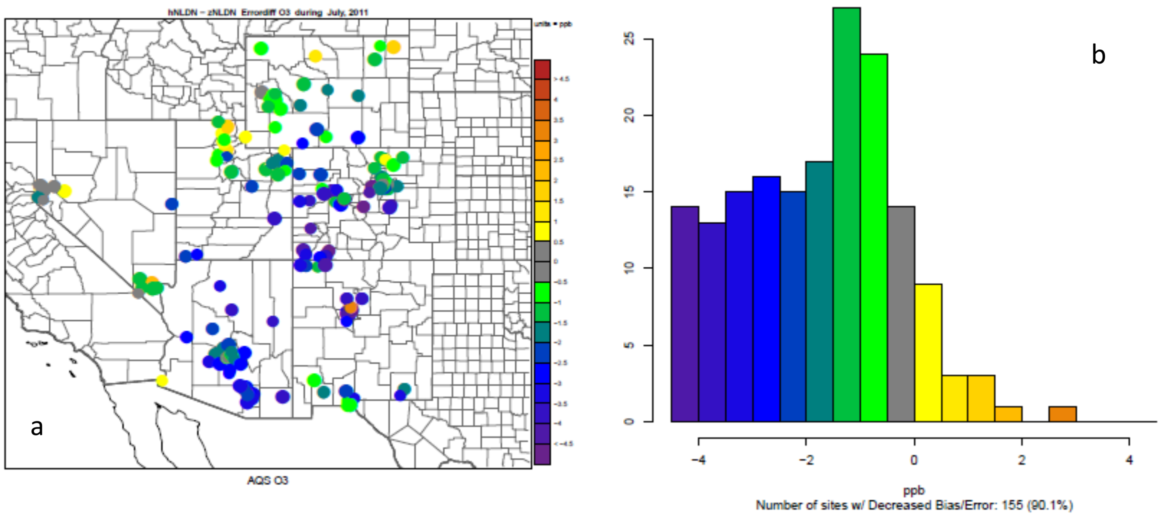

Supplementary Figure 2. Error difference (NLDN – Base) of DM8HR O_3 between the simulations with and without lightning NO_X. a. Spatial distributions over MWS. b. The histogram showing also the number of sites having reduced errors.

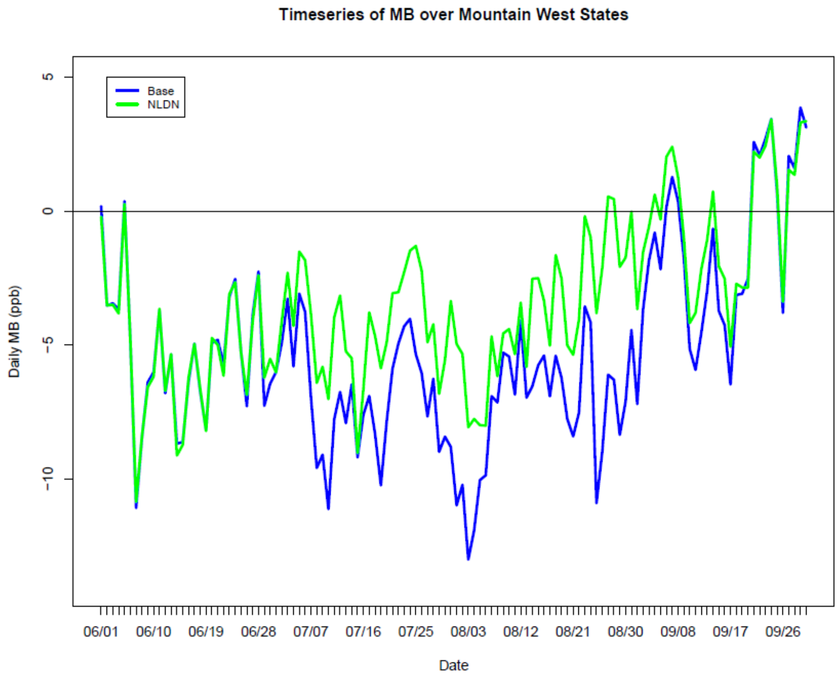

Supplementary Figure 3. The daily Mean Bias (MB, model – obs) of DM8HR O_3 averaged on the AQS sites in MWS for the Base (without lightning NO_x) and NLDN (with lightning NO_x) simulations.

As shown in Supplementary Figure 3 (corresponding to Figure 1e in the main text where the difference of MB between NLDN and Base is presented), the mean MB values over the AQS sites in MWS for both the Base and NLDN cases are mostly negative during the June to September period. With lightning NO_x, the NLDN case brings the MB values closer to the zero line when active lightning activities are present.

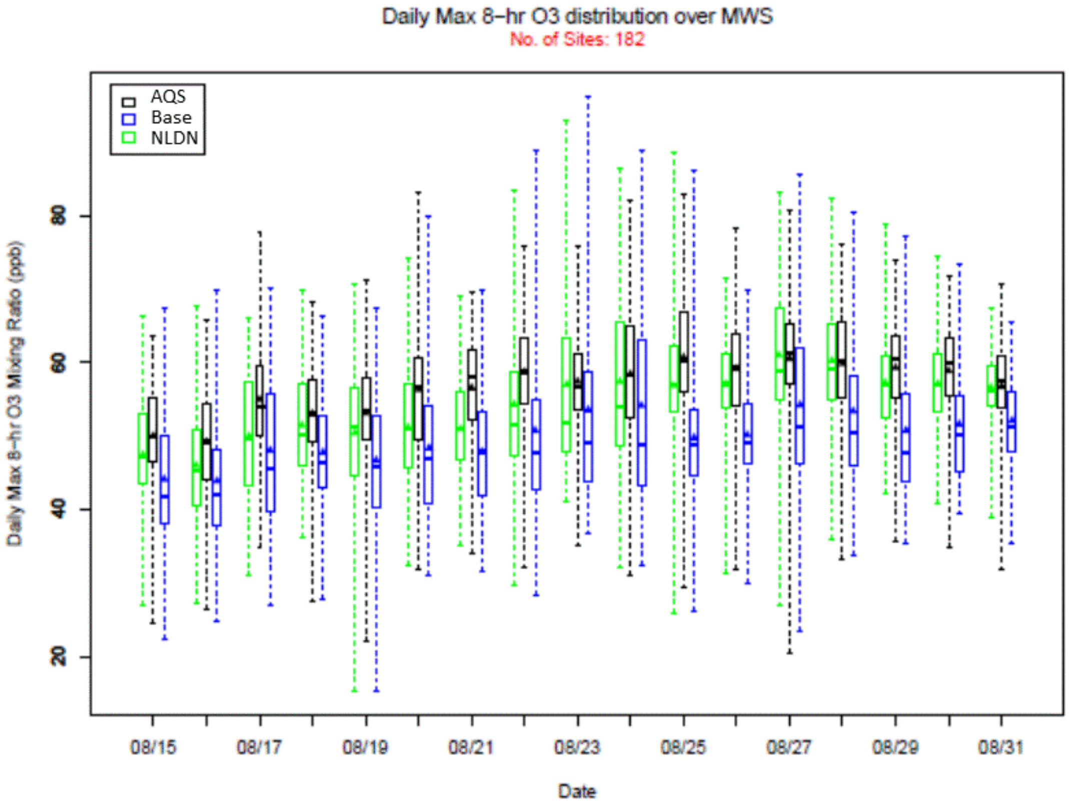

Supplementary Figure 4. Observed and simulated DM8HR O_3 mixing ratio distribution during the episode from August 15 to August 31 in MWS; the boxplots show the maximum, minimum, the 75th and 25th percentiles, median (rectangle), and mean (triangle).

Episodic DM8HR O_3 boxplots

Supplementary Figure 4 presents the observed and simulated DM8HR O_3 boxplots for the period from August 15th to August 31st at MWS showing the range of variations across the 182 AQS sites in the NWS region. The simulation with lightning NO_x results in a much better match with the observations than the simulation without lightning NO_x throughout this entire period.

Calculation of lightning flashes at an observation site

In Fig. 1e of the main text, we overlapped the time series of lightning flash counts with the time series of MB reduction between two simulations (NLDN and Base). Since the impact of lightning-induced NO_x on O_3 is nonlinear and can either destroy O_3 via titration or produce O_3 depending on the availability of other precursors and sunlight, the simple correlative relationship between the lightning flashes and O_3 mixing ratios should be viewed as qualitative indicators. For

each AQS site, we first locate the model grid cell where it resides, then we sum up the daily total lightning flashes that occurred in the grid cell and its surrounding grid cells. We have tested the number of the surrounding grid cells from one (9 grid cells including the one where the site is located corresponding to an area of 36x36 km^2) to the entire area, that is, all the sites have the same number of lightning flashes occurred during the day over the region. Supplementary Figure 5 shows the correlation coefficients between the lightning flashes summed over varying number of surrounding grid cells and the reduction of MB at AQS sites for the two model simulations. The correlation coefficient (absolute value; since the reduction of MB inversely correlates with the lightning flashes, the correlation coefficient is negative) initially increases with the increase of the number of surrounding grid cells, and it levels off at the maximum around 30 to 35 surrounding grid cells (corresponding to an area of ~800x800 km^2), then it starts to decline when the area is further expanded.

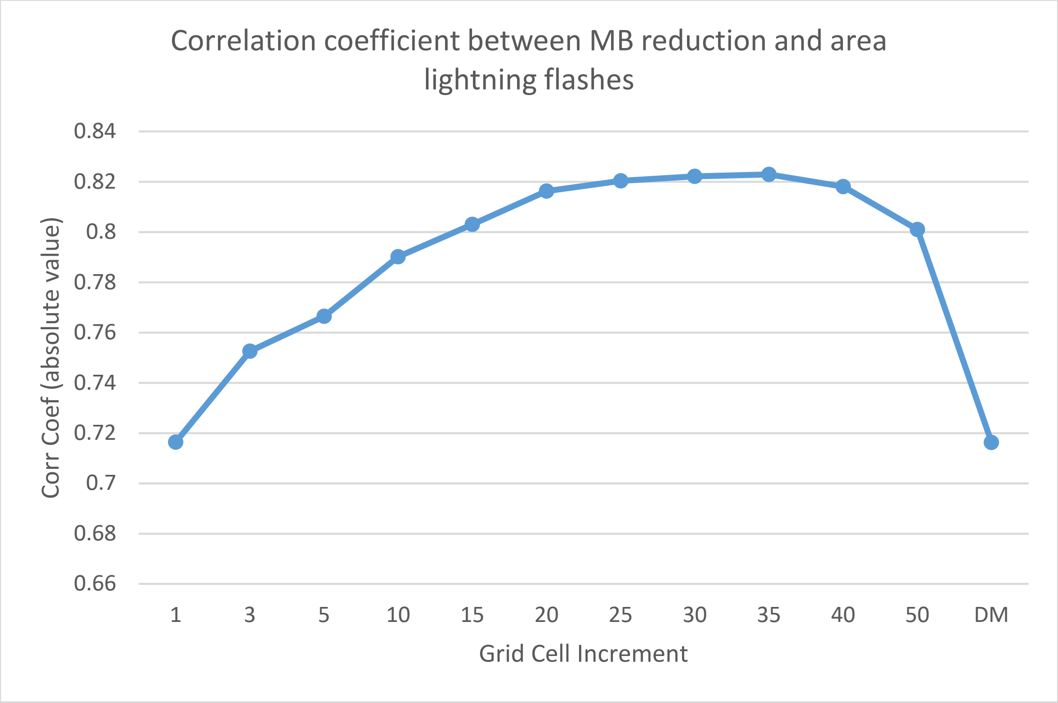

Supplementary Figure 5. Correlation coefficients between lightning flashes and the reduction of DM8HR O_3 MB for the simulations with and without lightning NO_X. DM: the entire area of MWS.

Lightning flashes and the corresponding NO emission across the years

In the main text, we presented the lightning flashes and the corresponding NO emissions over MWS for July from 2011-2016. Here in Supplementary Figure 6 and Supplementary Figure 7, the same are presented for August.

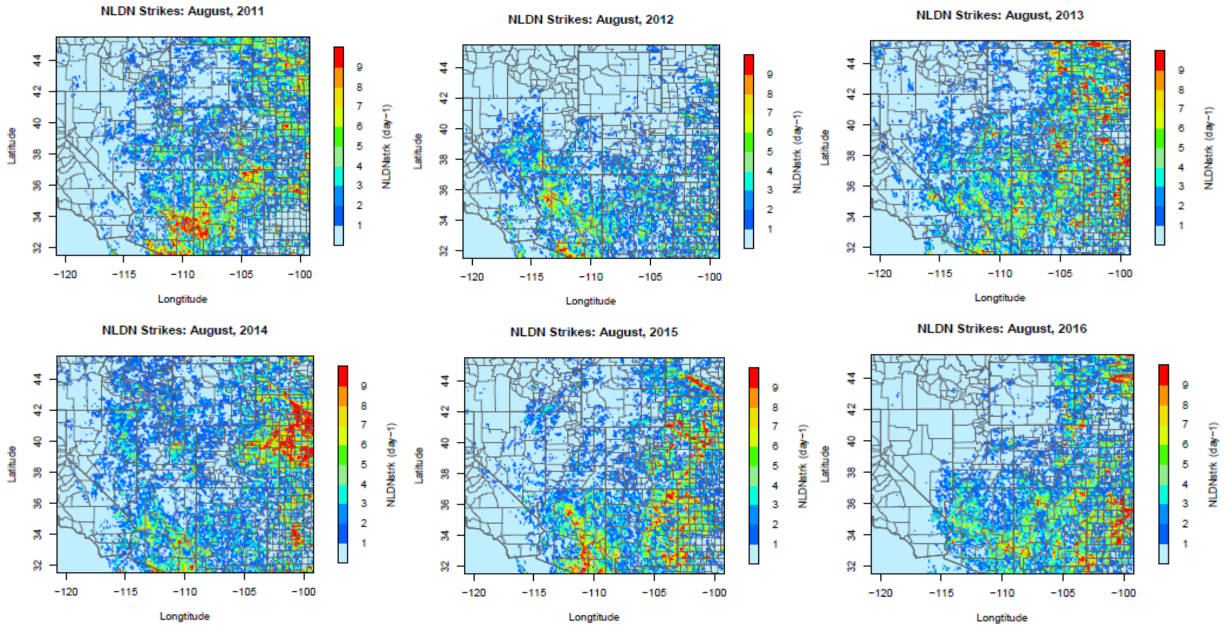

Supplementary Figure 6. Lightning flashes in August from 2011 to 2016 over MWS

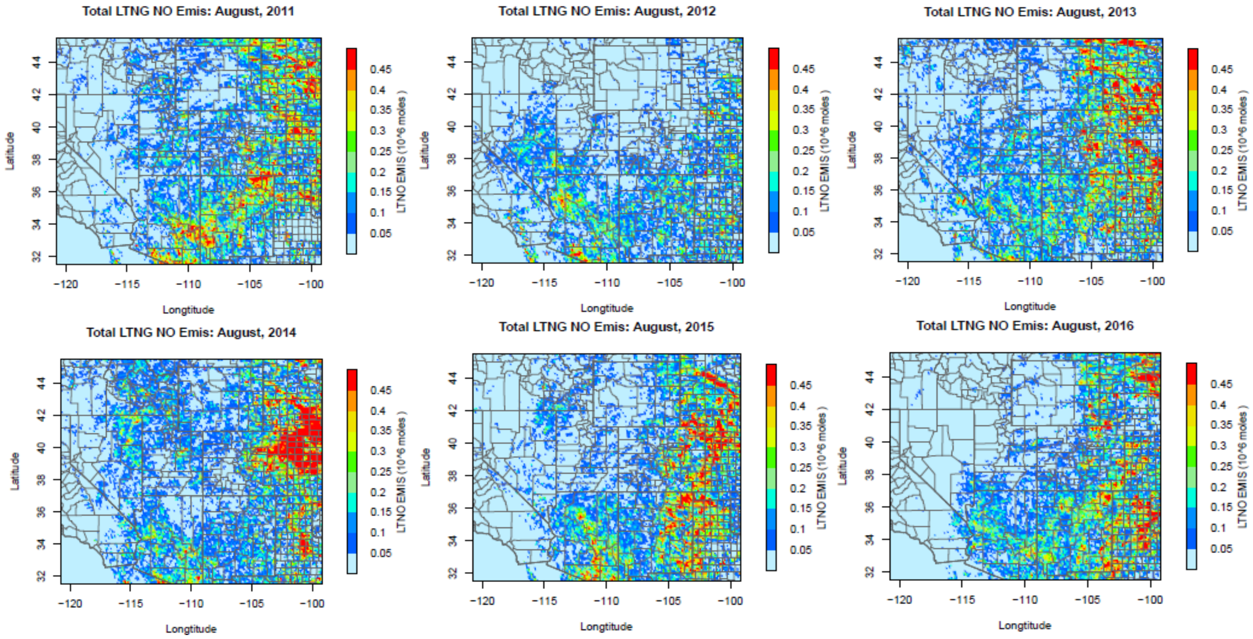

Supplementary Figure 7. Lightning-induced NO emissions in August from 2011 to 2016 over MWS

NO_X Budget in August

Supplementary Figure 8 presents the sources of NO_X emissions for August 2011 over MWS and Supplementary Figure 9 shows the relative daily contributions from each category and the daily percentage contribution of lightning NO to the total NO_X budget.

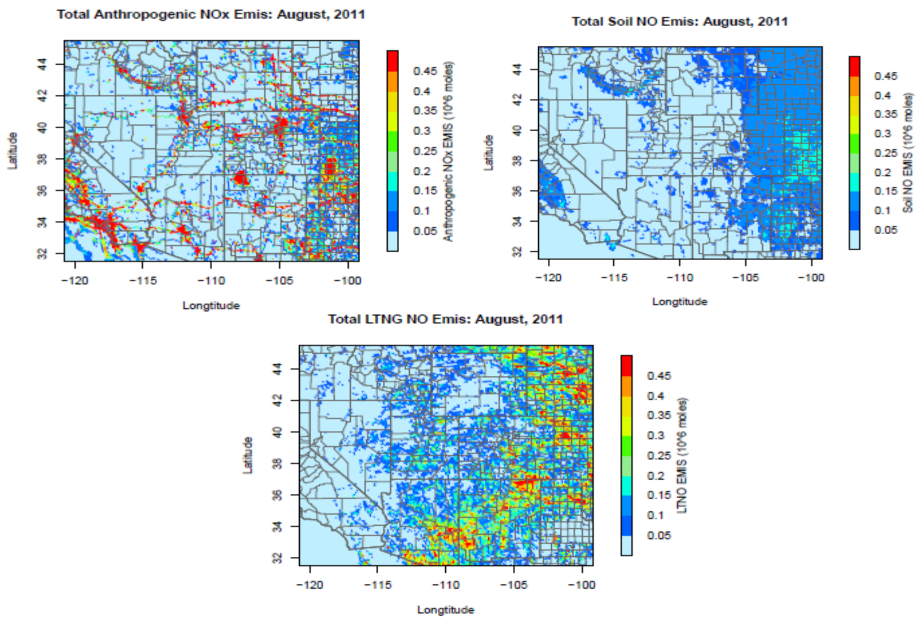

Supplementary Figure 8. The monthly total emissions from anthropogenic, soil, and lightning sources in August 2011.

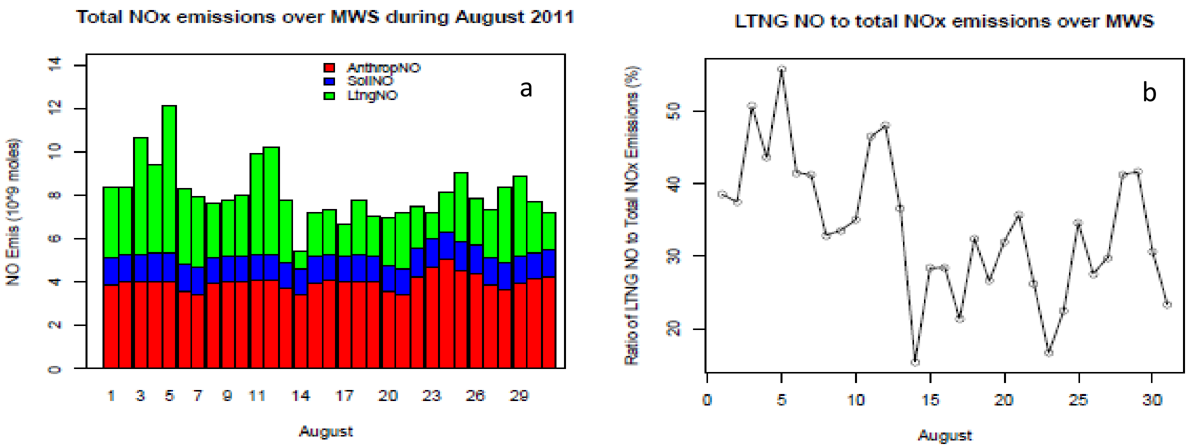

Supplementary Figure 9. Daily NO_X emissions from anthropogenic, soil, and lightning sources (a) and the daily percentage contribution of lightning NO to the total NO_X budget (b) in August at MWS.

Lightning Flashes surrounding BAO Site during July 26-29, 2014

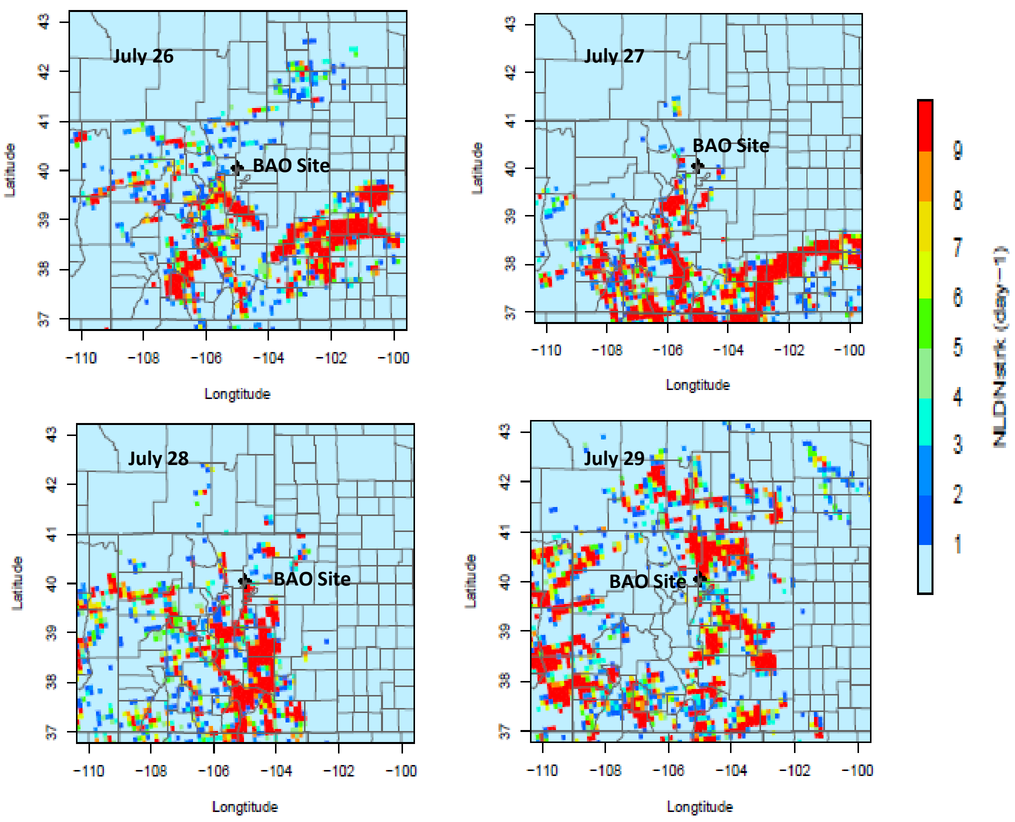

Supplementary Figure 10. Lightning flashes surrounding BAO Site during July 26-29, 2014

Supplementary Figure 10 shows the daily total lightning flashes at each 4x4 km grid cell from July 26-29, 2014 surrounding the BAO site where the Lidar measurements were performed.

Observed and Simulated Maximum O_3 Vertical Profiles

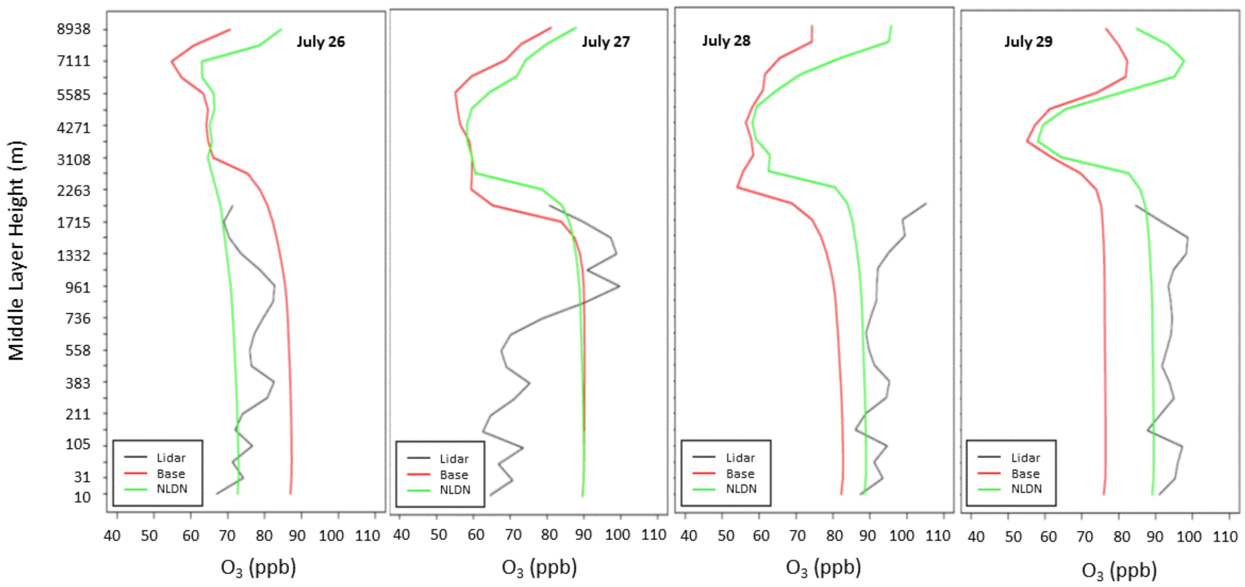

Supplementary Figure 11 was generated using the same data as in Fig. 3 except that the model layer was extended to Layer 30 to cover the tropopause region and it was plotted on the individual days to be easily discernible. It clearly indicates that the change of ground-level O_3 mixing ratios with NLDN was not the result of stratospheric intrusion.
